# Differences of genomic alterations and heavy metals in non-small cell lung cancer with different histological subtypes

**DOI:** 10.1007/s00432-023-04929-2

**Published:** 2023-05-31

**Authors:** Die Mu, Hui Tang, Gen Teng, Xinyang Li, Yarui Zhang, Ge Gao, Dongjuan Wang, Lu Bai, Xiangyao Lian, Ming Wen, Lisha Jiang, Shouxin Wu, Huihui Jiang, Cuimin Zhu

**Affiliations:** 1 Department of Oncology, Affiliated Hospital of Chengde Medical University, Chengde, 067000 China; 2 Shanghai Zhangjiang Institute of Medical Innovation, Shanghai Biotecan Pharmaceuticals Co., Ltd., Shanghai, 200135 China; 3 Department of Interventional and Vascular Surgery, Shanghai Tenth People’s Hospital, School of Medicine, Tongji University, Shanghai, 200072 China

**Keywords:** Lung adenocarcinoma, Lung squamous cell carcinoma, Tissue, Circulating tumor DNA, Genomic alterations, Heavy metals

## Abstract

**Purpose:**

This study aimed to explore the correlations among heavy metals concentration, histologic subtypes and molecular characteristics in patients with non-small cell lung cancer (NSCLC).

**Methods:**

In this study, an NGS panel of 82 tumor-associated genes was used to identify genomic alternations in 180 newly diagnosed patients with NSCLC. The concentrations of 18 heavy metals in the serum samples were detected by inductively coupled plasma emission spectrometry (ICP-MS).

**Results:**

A total of 243 somatic mutations of 25 mutant genes were identified in 115 of 148 patients with LUAD and 45 somatic mutations of 15 mutant genes were found in 24 of 32 patients with LUSC. The genomic alternations, somatic interactions, traditional serum biomarkers, and heavy metals were markedly different between patients with LUAD and LUSC. Moreover, patients with LUSC were significantly positively correlated with Ba, but not LUAD. Lastly, patients with *EGFR* mutations presented significant negative correlations with Cd and Sr, whereas patients with *TP53* mutations showed a significant positive correlation with Pb.

**Conclusion:**

The genomic alternations, somatic interactions, traditional serum biomarkers, and heavy metals were different between patients with LUAC and LUSC, and heavy metals (e.g., Ba, Pb, and Cd) may contribute to the tumorigenesis of NSCLC with different histological and molecular subtypes.

**Supplementary Information:**

The online version contains supplementary material available at 10.1007/s00432-023-04929-2.

## Introduction

With the development of personalized therapy, the sub-classification of NSCLC is ever more necessary. Previous studies have well indicated that lung adenocarcinoma (LUAD) and lung squamous cell carcinoma (LUSC) derived from distinct epithelial cells, exhibited distinct clinic-pathological features and expressed distinct biomarkers (Chen and Dhahbi 2021; Relli et al. 2019; Wang et al. 2022). Moreover, different genomic mutation landscapes between these two subtypes were also identified, which were higher mutation rates of *EGFR*, *ALK*, *RET*, *ROS1*, and *KRAS* in LUAD, and more prevalent mutations of *PIK3CA*, *AKT1*, and *CDKN2A* in LUSC (Campbell et al. 2016; Cancer Genome Atlas Research 2012, 2014). Especially, East Asian women without a smoking history tend to be developed LUAD and display a higher mutation rate of *EGFR* and a lower incidence of *KRAS* mutation (Ha et al. 2015; Li et al. 2011). What is more, prognosis also differs between these two subtypes (Wang et al. 2020). LUAD is more likely to develop in brain metastases after surgery and combined modality therapy compared with LUSC (Mamon et al. 2005; McAleese et al. 2019). Although the differences in clinic-pathological features, traditional serum biomarkers, and genomic profiles between LUAD and LUSC have been well studied, little is known about the difference in heavy metals.

Many studies have explored the association between environmental exposure to heavy metals (including As, Cd, Cr, Cu, Hg, Ni, Pb, and Zn) and lung cancer (Caffo et al. 2014; Hartwig et al. 2002; Huff et al. 2016; Kim et al. 2015). Metal dyshomeostasis could result in the activation of oncogenic signaling pathways, inhibition of the DNA repairing system, production of oxidative stress, and modification of epigenetic inheritance (Caffoet al. 2014; Hartwiget al. 2002; Huffet al. 2016; Kim et al. 2015). The concentration of Cu and the ratio of Cu/Zn derived from serum in patients with lung cancer were higher than that in healthy controls (Diez et al. 1989). The discharge amount of Zn was positively correlated with the incidence of lung cancer in Texas, USA (Coyle et al. 2006). Moreover, Huang et al. reported that the concentration of Cu in the soil is significantly positively correlated with LUAD for both sexes and with LUSC only for males (Huang et al. 2013). In addition to the effects of heavy metals on tumorigenesis, certain studies also focused on the relevance between heavy metals and prognosis. Low Cd levels in the blood (< 1.47 µg/L) were significantly correlated with improved overall survival in patients with lung cancer with stage IA (Pietrzak et al. 2021). In comparison with Fe-negative patients with LUAD, Fe-positive patients displayed improved overall survival accompanying higher numbers of M1-like pro-inflammatory tumor-associated macrophages (Thielmann et al. 2019). However, this unique influence of Fe on patients with LUSC was not observed (Thielmannet al. 2019). Collectively, both tumorigenic and prognostic effects of heavy metals showed specificities on different histological subtypes of NSCLC, whose elucidations might be useful for anti-cancer therapy.

In our study, targeted next-generation sequencing (NGS) was carried out to detect tissue DNA and ctDNA from NSCLC patients by an NGS panel of 82 tumor-associated genes. The differences in clinic-pathological features, traditional serum biomarkers, genomic alterations, somatic interactions, and the deposition of heavy metals were systematically compared between patients with LUAD and LUSC. Furthermore, we also investigated the correlations among histological subtypes, genomic alterations, traditional serum biomarkers, and heavy metals.

## Materials and methods

### Patients and samples collection

One hundred and eighty newly diagnosed patients with NSCLC from the Department of Oncology in the Affiliated Hospital of Chengde Medical University were recruited between April 2020 and July 2021. The pathological diagnosis was confirmed by three independent pulmonary pathologists based on the 4th edition of the World Health Organization Classification of Lung Tumors (Travis et al. 2015). Ninety-one Formalin-Fixed and Paraffin-Embedded (FFPE) tumor specimens and 89 plasma samples were collected for NGS analysis. This study was implemented according to the Code of Ethics of the World Medical Association (Declaration of Helsinki) (Gandevia and Tovell 1964).

### DNA extraction and quality control

The methods of DNA extraction and quality control were quoted from our published paper, which was described as follows: “Genomic (g) DNA from the FFPE tumor specimens was extracted by GeneRead DNA FFPE Kit (Qiagen, Hilden, Germany). The quantity and purity of gDNA were evaluated by Qubit^®^ 3.0 Fluorometer (Invitrogen, Carlsbad, CA, USA) and NanoDrop ND-1000 (Thermo Scientific, Wilmington, DE, USA). DNA Integrity was assessed by the Agilent 2100 Bioanalyzer instrument (Agilent Technologies) via the High Sensitivity DNA Reagent (Agilent Technologies, Santa Clara, CA, USA).”(Nian et al. 2022).

### Library preparation, hybridization capture, and illumina sequencing

Firstly, 300 nanogram gDNA per sample broke by an E220 focused ultrasonicator Covaris (Covaris, LLC.) to get 150–200 bp DNA fragments. Secondly, 10–100 nanogram DNA fragments were used for library construction by the KAPA library preparation kit (Kapa Biosystems Inc.; Roche Diagnostics). Thirdly, the NGS libraries were trapped by the xGen Lockdown Probe pool (Integrated DNA Technologies, Inc.), and the captured DNA fragments were amplified by 1X KAPA HiFi Hot Start Ready Mix (Kapa Biosystems Inc.; Roche Diagnostics). Lastly, the Illumina NextSeq CN500 platform with a medium flux chip (NextSeq CN500 Mid Output v2 kit; Illumina Inc.) was adopted to establish the NGS libraries (Nian et al. 2022).

### Bioinformatics analysis

The methods of bioinformatics analysis were quoted from our published paper, which was described as follows: “The low-quality reads were filtered to gain clean data. All filtered reads were aligned to the human genome (University of California Santa Cruz ID: hg19) via the Burrows-Wheeler Aligner v. 0.7.12. Then, the Picard and Genome Analysis Toolkit (GATK v.3.2) method was adopted for duplicate removal, local realignment, and base quality score recalibration, which was also used to generate the quality statistics. Lastly, the VarDict was used for the authentication of single nucleotide variation (SNV) and Insertion/Deletion (InDel).

The ANNOVAR software tool was adopted for annotating somatic mutations. The candidates of somatic mutations were recognized by the following filter conditions: (i) remove mutations with coverage depth (VDP) less than 10×; (ii) remove variant sites with mutant allele frequency (MAF) > 0.001 in the 1000 Genomes databases (1,000 Genomes Project Consortium; https://www.internationalgenome.org/); (iii) retain variant sites with MAF ≥ 0.001 and < 0.1 in the 1000 Genomes databases with COSMIC evidence (http://cancer.sanger.ac.uk/cosmic); (iv) retain variations in the exonic or splicing region (10 bp upstream and downstream of splicing sites); (v) remove synonymous mutations; (vi) remove unknown variant classification; and (vii) remove the functional benign variant sites predicted by MutationTaster, PolyPhen-2 or SIFT. The biological consequences were explored by the Kyoto Encyclopedia Of Genes and Genomes (KEGG) and Gene Ontology (GO) enrichment analysis via the cluster Profiler package (http://bioconductor.org/packages/release/bioc/html/ clusterProfiler.Html) (Yu et al. 2012). The correlation between the genomic alternations and their clinical consequences was defined by OncoKB Precision Oncology Database (http://oncokb.org/).”(Nian et al. 2022).

### Traditional serum biomarker detection

At least 10 mL of whole blood for each patient was centrifuged at 3000 rpm/10 min at 4 °C to get the upper serum. The buffer solution (PH = 7.5) was used to prepare the standard solutions of carbohydrate antigen 125 (CA125), carcinoembryonic antigen (CEA), cytokeratin-19 fragments (CYFRA21-1), neuron-specific enolase (NSE), progesterone-releasing peptide (ProGRP), and squamous cell carcinoma antigen (SCC) adopting the serial dilution method. The levels of these traditional serum biomarkers were detected by chemiluminescence immunoassay (Gong and Zhang 2020; Qu et al. 2013). The testing equipment was an automated chemiluminescence immunoassay analyzer (COBAS 8000 E 801; Roche Diagnostics GmbH), and the detection kits of CA125, CEA, CYFRA21-1, NSE, ProGRP, and SCC were all originally imported from the Roche Diagnostics GmbH.

### ICP-MS detection

The concentrations of 18 heavy metals in serum samples were detected by inductively coupled plasma mass spectrometry (ICP-MS) (Agilent 7800), including Arsenic (As), Barium (Ba), Cadmium (Cd), Cobalt (Co), Chromium (Cr), Cuprum (Cu), Gallium (Ga), Mercury (Hg), Manganese (Mn), Nickel (Ni), Plumbum (Pb), Stibium (Sb), Selenium (Se), Stannum (Sn), Strontium (Sr), Thallium (Tl), Vanadium (V), and Zinc (Zn). ICP-MS is used for multi-elemental capabilities analysis and the detection procedures are detailed in the manufacturer’s instructions (Zhao et al. 2018). In simple terms, at least 2 ml of whole blood for each patient was centrifuged at 3000 rpm/10 min to get the upper serum. The centrifugal serum was stored at − 20 °C.

### Statistical analysis

The maftools package was adopted to depict genomic landscapes, lollipop plots, and spectrums of co-occurring and mutually exclusive genomic alterations via R software (R 4.0.3, R Core Team; https://www.R-Project.org). Fisher’s exact test was used to evaluate the statistical differences in categorical variables between the patients with LUAD and the patients with LUSC by R software. Continuous variables are shown as median with interquartile range (IQR) and they were compared by Mann–Whitney U test between the two groups in GraphPad Prism (v.7.0; GraphPad Software, La Jolla, CA). Correlation analysis among histologic subtypes, genomic alternations, 6 traditional serum biomarkers, and 18 heavy metals was conducted by Spearman’s Rank Correlation Analysis. A *P* value of less than 0.05 was considered statistically significant.

## Results

### Patient characteristics

In this study, the number of newly diagnosed NSCLC patients with stages I, II, III, and IV was 0, 1, 21, and 158, respectively. In terms of histologic type, 148 patients were diagnosed with LUAD, and 32 patients were LUSC. One hundred and seventy-six patients were detected by both heavy metals and traditional serum biomarkers. Significant differences in gender, *EGFR* mutation, smoking history, anatomical staging, brain metastatic status, CEA level, and SCC level were found between patients with LUAD and LUSC, whereas no significant differences were observed in other demographic and clinical characteristics, including age, height, weight, BMI, *TP53* mutation, the history of drinking, CA125 level, CYFRA21-1 level, NSE level, and ProGRP level (Table 1).

**Table 1:** The clinical characteristics of patients according to histologic subtypes

Clinical characteristics	No. of patients	Histologic subtypes		*P* value
		LUAD	LUSC	
Total Sample	180	148	32	
Gender				
Male	100	76	24	0.011*
Female	80	72	8	
EGFR mutation				
Yes	75	72	3	0.000****
No	105	76	29	
TP53 mutation				
Yes	91	72	19	0.183
No	89	76	13	
Smoking history				
Once	29	21	8	0.001**
Now	41	27	14	
Never	110	100	10	
Drinking history				
Once	13	11	2	0.096
Now	28	19	9	
Never	139	118	21	
Anatomical staging				
I	0	0	0	0****
II	1	1	0	
III	21	7	14	
IV	158	140	18	
Brain metastasis				
Yes	70	65	5	0.002**
No	110	83	27	
CA125 at baseline				
Normal	78	68	10	0.098
Elevated	98	77	21	
Unknown	4	3	1	
CEA at baseline				
Normal	61	45	16	0.026*
Elevated	115	100	15	
Unknown	4	3	1	
CYFRA21-1 at baseline				
Normal	58	50	8	0.238
Elevated	118	95	23	
Unknown	4	3	1	
NSE at baseline				
Normal	91	77	14	0.272
Elevated	85	68	17	
Unknown	4	3	1	
ProGRP at baseline				
Normal	159	129	30	0.158
Elevated	17	16	1	
Unknown	4	3	1	
SCC at baseline				
Normal	149	129	20	0.002**
Elevated	27	16	11	
Unknown	4	3	1	
Age (median with IQR)	59 (54.25–64)	59 (54.25–65)	59.5 (54.25–62.75)	
Height/m (median with IQR)	1.65 (1.59–1.7)	1.63 (1.58–1.70)	1.67 (1.62–1.71)	
Weight/Kg (median with IQR)	62.25 (56–70)	62 (55.63–70)	67.25 (58.25–72)	
BMI (median with IQR)	23.48 (21.09–25.38)	23.54 (21.09–25.35)	23.4 (21.09–26.03)	

*P < 0.05, **P < 0.01, ****P < 0.05

### Differences in genomic alterations between patients with LUAD and LUSC

To delineate the landscapes of genomic alterations, the somatic mutations from the tumor tissue DNA of 91 patients and the plasma-derived ctDNA of 89 patients were analyzed by an NGS panel of 82 tumor-associated genes (Table S1). We mainly focused on protein-altering variants according to the annotation of somatic SNVs and InDels. A total of 195 somatic variants of 25 mutated genes were detected in 86 out of 91 (94.51%) tumor tissues (Supplementary Fig. 1A and Table S2), and 93 somatic variants of 17 mutated genes were identified in 54 out of 89 (60.67%) peripheral blood samples (Supplementary Fig. 1B and Table S2). Our results were consistent with previous studies that the rate of mutation detection from tumor tissue DNA was much higher than that from plasma-derived ctDNA (Cai et al. 2021). Moreover, the percentages of tissue DNA-specific identified genes, ctDNA-specific identified genes, and co-identified mutated genes were 45.16% (14/31), 19.35% (6/31), and 35.48% (11/31) in NSCLC, respectively (Supplementary Fig. 1C).

**Fig. 1:**
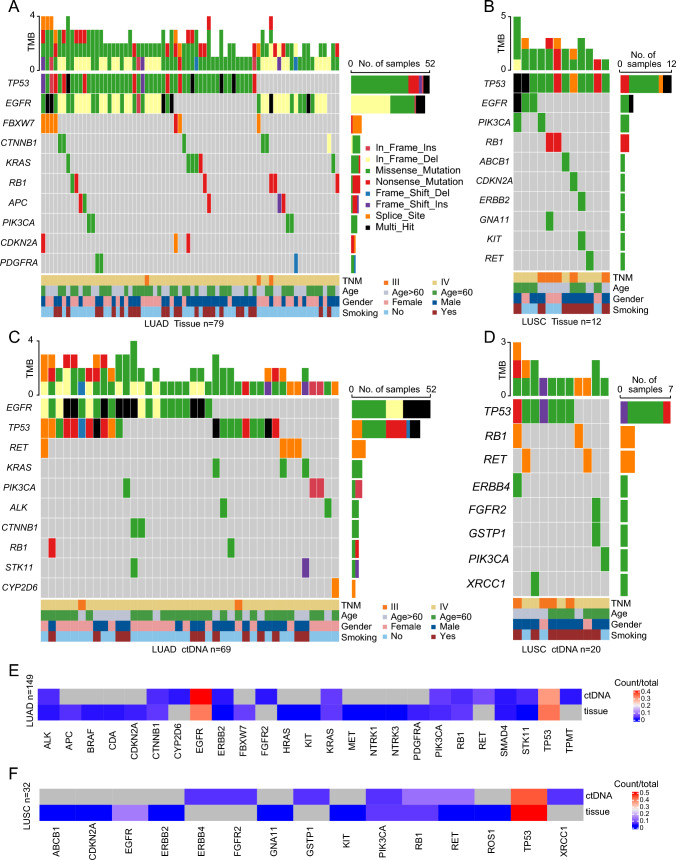
Somatic mutation landscape of NSCLC derived from LUAD tumor tissue DNA (*n* = 79) (**A**), LUSC tumor tissue DNA (*n* = 12) (**B**), LUAD ctDNA (*n* = 69) (**C**), and LUSC ctDNA (*n* = 20) (**D**). Patients were arranged along the x-axis. Mutant genes are ranked by mutant frequency and the right panel shows the number of samples with nonsynonymous mutations. Tumor mutation burden (TMB, mutations per Mb) is shown in the upper panel. Concordance of mutated genes between tumor tissue DNA and ctDNA in patients with LUAD (**E**) and LUSC (**F**)

It is well established that NSCLC patients carrying mutations of driver genes benefit much from the targeted therapies, and different genomic profiles have been uncovered between LUAD and LUSC (Campbellet al. 2016; Cancer Genome Atlas Research 2012, 2014). In this study, 243 somatic mutations of 25 mutant genes were identified in 115 of 148 (77.70%) patients with LUAD (Fig. 1A, C, and Table S3), and 45 somatic mutations of 15 mutant genes were found in 24 of 32 (75.00%) patients with LUSC (Fig. 1B, D, and Table S3). Among these mutant genes, *ABCB1*, *ERBB4*, *GNA11*, *GSTP1*, *ROS1*, and *XRCC1* were exclusively identified in patients with LUSC, whereas 16 mutant genes were uniquely present in patients with LUAD. Nine mutant genes (*CDKN2A*, *EGFR*, *ERBB2*, *FGFR2*, *KIT*, *PIK3CA*, *RB1*, *RET*, and *TP53*) were concurrently detected in both groups. To further compare the feasibility of genomic profiling of patients with different histologic subtypes using plasma-derived ctDNA, the consistencies of genomic profiling between tissue DNA and ctDNA in the LUAD group and the LUSC group were all analyzed by the above NGS panel. Consistent with previous studies (Cai et al. 2021), ctDNA analysis has a higher consistency of genomic profiling in patients with LUAD (40.00%, 10/25) than that in patients with LUSC (26.67%, 4/15) (Fig. 1E and F).

Furthermore, we also found that the frequencies and sites of these concurrent mutant genes between patients with LUAD and with LUSC were markedly different (Supplementary Fig. 2 and Table S4). For example, although about half of the patients carried *TP53* mutations in both LUAD (48.65%, 72/148) and LUSC (59.38%, 19/32) groups, marked differences in the mutant types and sites were observed (Supplementary Fig. 2A). The in-frame insertion and frame-shift deletion of *TP53* were exclusively identified in the LUAD group, whereas Q60fs, K93N, G113R, and other nine mutant sites were specifically observed in the LUSC group.

### Differences in somatic interactions between patients with LUAD and LUSC

**Fig. 2:**
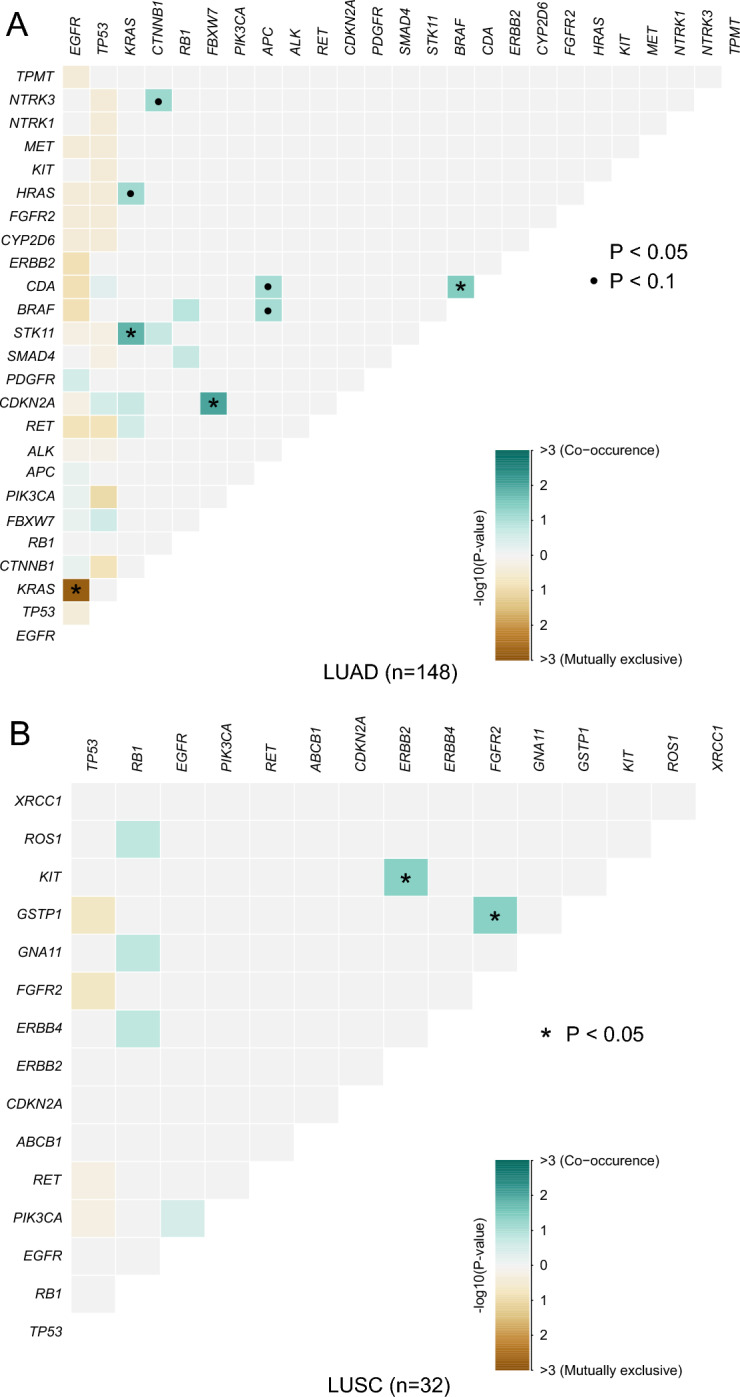
The spectrum of co-occurring and mutually exclusive genomic alterations in patients with LUAD (**A**) and LUSC (**B**)

Patients with LUAD showed a higher mutation rate of *EGFR* compared to those with LUSC. Interestingly, *EGFR* mutations and Kirsten rat sarcoma (*KRAS*) mutations are generally mutually exclusive, and patients with both mutations confer resistance to EGFR-TKIs (Pao et al. 2005). In our study, the somatic interactions observed in patients with LUAD were markedly different from those with LUSC. *EGFR* and *KRAS* were mutually exclusive in the LUAD group (*P* = 0.0016) (Fig. 2A and Table S5), whereas no mutually exclusive interactions were found in the LUSC group (Fig. 2B and Table S5). Meanwhile, marked differences in the co-occurring set of genes were also identified between these two groups. *FBXW7 *and *CDKN2A* (*P* = 0.0093), *KRAS* and *STK11* (*P* = 0.0158), and *CDA* and *BRAF* (*P* = 0.0346) were significant co-occurring pairs of genes in patients with LUAD (Fig. 2A and Table S5), while *KIT* and *ERBB2* (*P* = 0.0417), and *GSTP1* and *FGFR2* (*P* = 0.0417) were observed in patients with LUSC (Fig. 2B and Table S5).

### Differences in traditional serum biomarkers and heavy metals between patients with LUAD and LUSC

To further explore the differences in NSCLC with different histologic subtypes, we first compared the levels of six traditional serum biomarkers between patients with LUAD (*n* = 148) and with LUSC (*n* = 32). Expectedly, the levels of CEA and SCC were significantly different between the LUAD group and the LUSC group, and their median concentrations with IQR were as follows: CEA 13.21 (3.75–65.58) vs. 4.3 (2.36–16.15) ng/mL, and SCC 0.82 (0.51–1.38) vs. 1.21 (0.81–8.31) ng/mL (Supplementary Fig. 3 and Table S6).

**Fig. 3:**
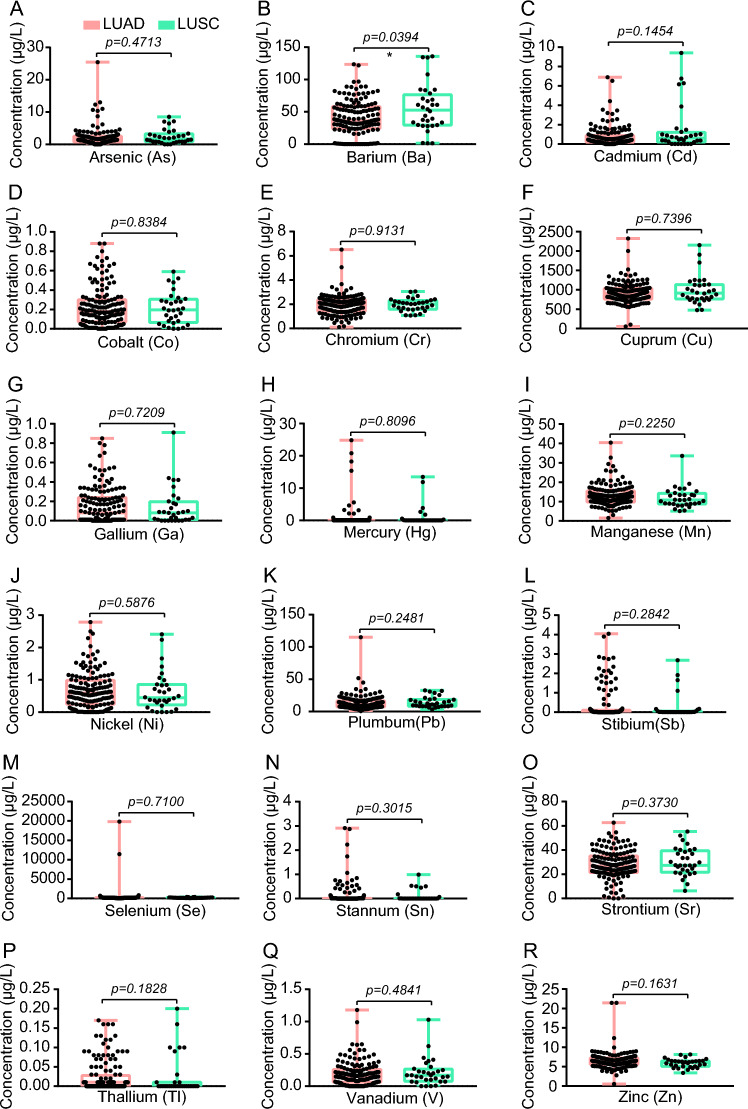
The comparative analysis of 18 heavy metals between patients with LUAD and LUSC. Statistical analysis was performed by the two-tailed Mann–Whitney *U* test. *P < 0.05

Next, we compared the concentration of the 18 heavy metals between these 2 groups, and only Ba showed significant differences (Fig. 3 and Table S6). The median concentrations with IQR for Ba in the LUAD group and the LUSC group were as follows: 36.27 (24.18–57.73) vs. 50.88 (29.62–78.54) μg/L (Fig. 3B).

### Differences in traditional serum biomarkers and heavy metals between LUAD patients with and without *EGFR* mutations

**Fig. 4:**
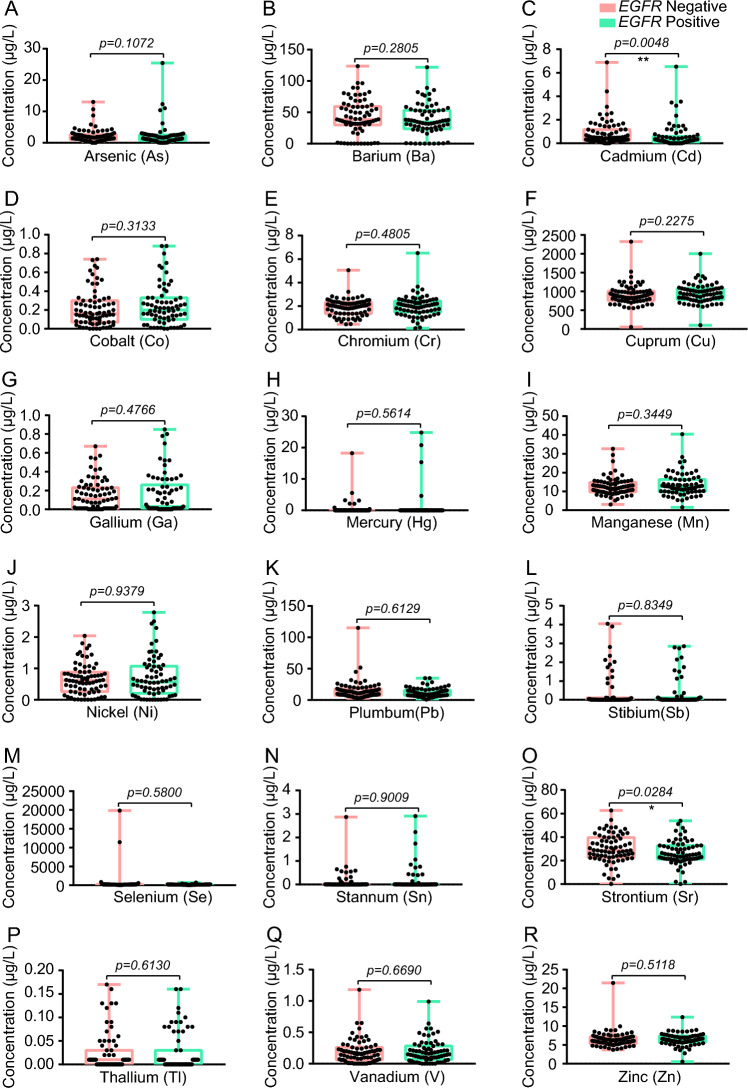
The comparative analysis of 18 heavy metals between LUAD patients with and without *EGFR* mutations. Statistical analysis was performed by the two-tailed Mann–Whitney U test. *P < 0.05, **P < 0.01

To further complement the biological characteristics of LUAD in terms of 6 traditional serum biomarkers and 18 heavy metals, we performed comparative analyses between LUAD patients with (*n* = 70) and without *EGFR* mutations (*n* = 75). Interestingly, the levels of CYFRA21-1 and NSE in the *EGFR* positive group were all much higher than that in the *EGFR* negative group, whose median concentrations with IQR were as follows: CYFRA21-1 6.05 (3.505–10.08) vs. 4.12 (2.41–7.14) ng/mL, and NSE 18.34 (13.59–24.32) vs. 14.64 (12.13–19.22) ng/mL (Supplementary Fig. 4 and Table S6). Meanwhile, compared with patients without *EGFR* mutations, patients carrying *EGFR* mutations showed lower concentrations of Cd and Sr, and their median concentrations with IQR were as follows: Cd 0.29 (0.11–0.59) vs. 0.48 (0.26–1.17) μg/L, and Sr 24.78 (21.21–32.69) vs. 28.56 (22.63–39.58) μg/L (Fig. 4 and Table S6).

### Correlations analysis among histologic subtypes, genomic alternations, demographic and clinical characteristics, traditional serum biomarkers, and heavy metals

**Fig. 5:**
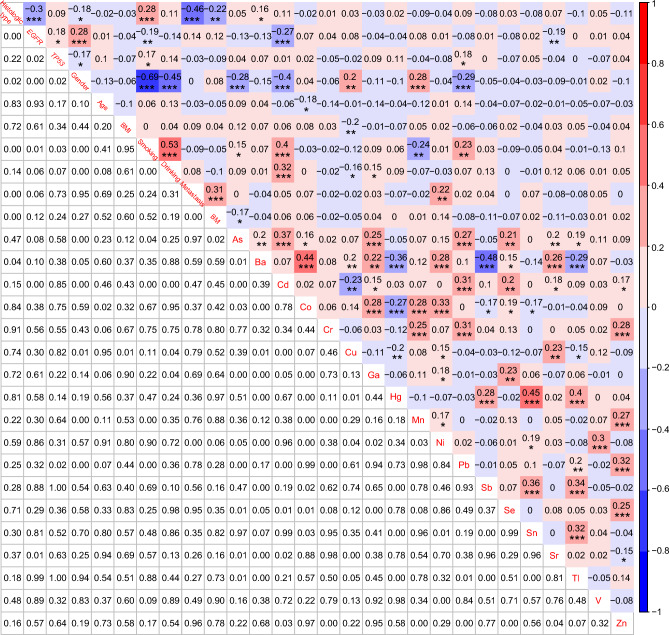
Correlations among histologic subtypes, *EGFR* mutations, *TP53* mutations, 7 demographic and clinical characteristics, and 18 heavy metals. *P < 0.05, **P < 0.01, and ***P < 0.001

To get the correlations among histologic subtypes, *EGFR* mutations, *TP53* mutations, 5 demographic characteristics, distant metastatic status, brain metastatic status, and 18 heavy metals, the NGS analysis and the heavy metal detection were simultaneously performed on 145 patients with LUAD and 31 patients with LUSC. LUSC was significantly negatively correlated with *EGFR* mutations (*r* = − 0.3, *P* < 0.001), Female (*r* = − 0.18, *P* < 0.05), distant metastatic status (*r* = − 0.46, *P* < 0.001), and brain metastatic status (*r* = − 0.22, *P* < 0.01), and it was also significantly positively correlated with smoking history (*r* = 0.28, *P* < 0.001). No significant correlations were found between the LUSC and the 18 heavy metals except Ba (*r* = 0.16, *P* < 0.05) (Fig. 5). Interestingly, the *EGFR* mutations presented a prominent negative correlation with Cd (*r* = − 0.27, *P* < 0.001) and Sr (*r* = − 0.19, *P* < 0.01), whereas noteworthy positive correlations were also uncovered between the *TP53* mutations and Pb (*r* = 0.18, *P* < 0.05) (Fig. 5). What is more, the concentration of Cd was significantly negatively correlated with females (*r* = − 0.4, *P* < 0.001), and significantly positively correlated with smoking history (*r* = 0.4, *P* < 0.001) and drinking history (*r* = 0.32, *P* < 0.001). Generally, our results were consistent with the previous finding that LUAD was more likely to occur in Chinese women carrying EGFR mutations without a history of smoking and drinking (Zhang et al. 2019). Lastly, significant correlations were also identified between most of the 18 heavy metals, such as As and Cd (*r* = 0.37, *P* < 0.001), As and Ga (*r* = 0.25, *P* < 0.001), As and Pb (*r* = 0.27, *P* < 0.001), and so on (Fig. 5).

**Fig. 6:**
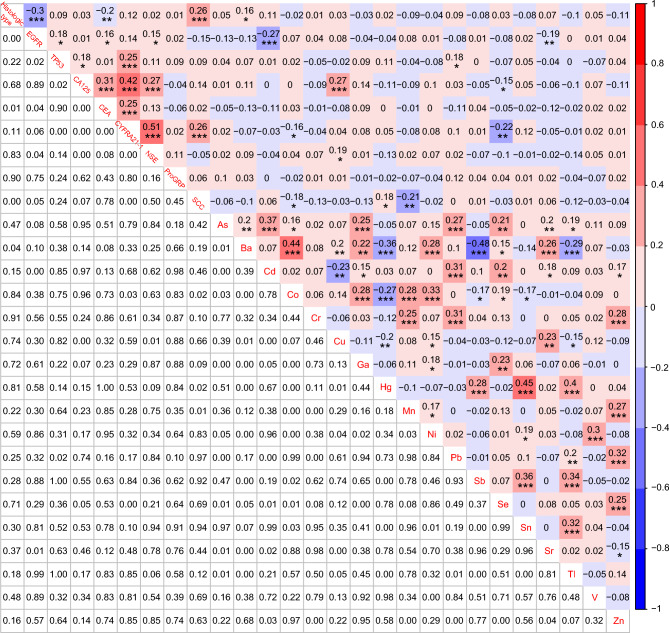
Correlations among histologic subtypes, *EGFR* mutations, *TP53* mutations, 6 traditional serum biomarkers, and 18 heavy metals. *P < 0.05, **P < 0.01, and ***P < 0.001

Although CA125, CEA, CYFRA21-1, and SCC have been studied well in NSCLC, little is known about their diagnostic values combined with heavy metals in different histological and molecular subtypes. Thus, we explored the correlations among histologic types, *EGFR* mutations, *TP53* mutations, 6 traditional serum biomarkers, and 18 heavy metals in 176 NSCLC patients. *EGFR* mutations were significantly positively correlated with CEA (*r* = 0.16, *P* < 0.05), and NSE (*r* = 0.15, *P* < 0.05), while *TP53* mutations were significantly positively correlated with CA125 (*r* = 0.18, *P* < 0.05) and CYFRA21-1 (*r* = 0.25, *P* < 0.001). Furthermore, we also observed significant positive correlations between CA125 and Cu (*r* = 0.27, *P* < 0.001), NSE and Cu (*r* = 0.19, *P* < 0.05), SCC and Hg (*r* = 0.18, *P* < 0.05), and significant negative correlations between CA125 and Se (*r* = − 0.15, *P* < 0.05), CYFRA21-1 and Co (*r* = − 0.16, *P* < 0.05), CYFRA21-1 and Se (*r* = − 0.22, *P* < 0.01), SCC and Co (*r* = − 0.18, *P* < 0.05), and SCC and Mn (*r* =− 0.15, *P* < 0.01) (Fig. 6).

## Discussion

The clinical management of patients with NSCLC is highly dependent on histologic types and molecular characteristics. Here, 148 LUAD patients and 32 LUSC patients were recruited in this study, and distinct characteristics in terms of gender, smoking history, anatomical staging, brain metastatic status, CEA level, and SCC level were found between these two groups (Relliet al. 2019). Two hundred and forty-three somatic mutations of 25 mutant genes were identified in 115 of 148 (77.70%) patients with LUAD and 45 somatic mutations of 15 mutant genes were found in 24 of 32 (75.00%) patients with LUSC. The genomic alternations, somatic interactions, traditional serum biomarkers, and heavy metals were markedly different between these two groups, further indicating that LUAD and LUSC should be classified and treated as different clinical entities. What is more, except for a significant positive correlation between LUSC and Ba, patients with *EGFR* mutations presented significantly negative correlations with Cd and Sr, and patients with *TP53* mutations showed a significant positive correlation with Pb, suggesting that heavy metals may be regarded as supplementary characteristics for the sub-classification of NSCLC with different histological and molecular subtypes. To our knowledge, this is the first time to report these above results in the East Asian population.

NSCLC is highly heterogeneous, and different clinic-pathological features, serum biochemical markers, and genomic profiles have been identified between LUAD and LUSC (Campbellet al. 2016; Chen and Dhahbi 2021; Chen et al. 2014; Wanget al. 2020). Consistent with previous studies (Kawase et al. 2012; Wanget al. 2020; Wang et al. 2022), more males, smokers, and patients with the III stage were diagnosed with LUSC compared to patients with LUAD. Moreover, cases with LUSC have a more frequent high level of SCC than patients with LUAD (Yu et al. 2021). However, one of our findings was inconsistent with the previous reports, which was patients with LUAD were more frequently diagnosed with stage IV rather than other stages, partly due to the patients from different departments and regions (Wanget al. 2020; Wang et al. 2022).

Expectedly, the mutation rate of *EGFR* in the East Asian population with LUAD was markedly higher (48.65%, 72/148) compared with the mutated frequency observed in the Western population (14–21%) (Cancer Genome Atlas Research 2014; Keedy et al. 2011; Zhanget al. 2019). Despite a higher mutation rate of *EGFR* occurring in non-smoking women (62.5%) compared with non-smoking men (28.57%) in LUAD, the mutation rate in non-smoking women with LUAD in this study (62.5%) was lower than these women in the CHOICE study (75%) (Zhanget al. 2019). The mutant status of *EGFR* was independently associated with LUAD and the history of smoking, but not with the gender of females (Kosaka et al. 2004; Wei et al. 2016). This is partly because of the high percentage of non-smokers but cooks among Asian women. Consistent with the previous studies, the mutation rates of *BRAF* (1.35%, 2/148) and *KRAS* (6.08%, 9/148) in LUAD were relatively lower in comparison with the Western population (*BRAF*: 2–10%, *KRAS*: 33–38%) (Cancer Genome Atlas Research 2014; Kinno et al. 2014; Myers et al. 2015; Schmid et al. 2009). In addition, distinct somatic interactions were also observed between LUAD and LUSC in the Chinese population. *FBXW7* and *CDKN2A*, *KRAS* and *STK11*, and *CDA* and *BRAF* were the only significant co-occurring pair of genes in patients with LUAD, while *KIT* and *ERBB2*, and *GSTP1* and *FGFR2* were exclusively observed in patients with LUSC. Genomic alterations in *KRAS* itself indicated the most common tumorigenic mutations in NSCLC, which was accompanied by a heterogeneous pattern of somatic interactions (Scheffler et al. 2019). In the LUAD patients with *KRAS* mutations, the most dominant co-occurring mutation was *STK11* in this study, whereas co-alterations in *TP53* were the biggest cluster in the Western population (Skoulidis and Heymach 2019).

Numerous studies have reported the close associations between NSCLC and environmental exposure to heavy metals, but few studies have focused on pathological cell types. Huang et al. reported that the concentration of Cu in the soil is significantly positively correlated with LUAD for both sexes and with LUSC only for males (Huanget al. 2013). Correspondingly, both the concentration of ceruloplasmin and the ratio of Cu/Zn in the serum of patients with lung cancer were much higher than those of healthy controls (Andrews 1979; Diezet al. 1989; Linder et al. 1981). However, the concentration of Cu in our study has not shown any significant differences between LUAD and LUSC, partly due to the limited number of LUSC and the regional divergence among different studies. Since the prognosis of patients with NSCLC is highly dependent on molecular characteristics, we also found a significant positive correlation between *TP53* mutations and Pb, and significant negative correlations between *EGFR* mutations and Cd and Sr. However, there are no reports about these findings yet, and these associations need to be further studied.

Ba is regarded as a low-toxic element, but its abnormal deposition was founded in multiple cancers (National Toxicology 1994). Yasemin et al. have reported that the concentration of Ba in the hair of patients with breast cancer is significantly lower than that of healthy controls (Benderli Cihan et al. 2011). 2.5–5 uM of Ba has independent abilities to promote anchorage-independent growth and/or invasion of keratinocytes and fibroblast (Thang et al. 2011), which was the precancerous hallmark of transformed cells (Bertotti et al. 2006; Li et al. 2008). However, no studies have reported that the concentration of Ba in patients with LUSC is significantly higher than that in patients with LUAD, which was the first time reported in the Chinese population. What is more, the interactions among different heavy metals are also important for tumorigenesis. The antineoplastic effects of As main reliance on the apoptosis of squamous cell carcinomas via producing reactive oxygen species (ROS) and activating JNK1/2 and caspase-3 (Eguchi et al. 2011; Potin et al. 2007), barium-mediated inhibition of arsenic-induced apoptosis can accelerate tumor progression in patients who exposure to both As and Ba (Yajima et al. 2012). In our study, significant correlations were observed between most of the 18 heavy metals, including As and Ba. Thus, it is interesting to explore the activation and/or inhibition of molecular mechanisms underlying these heavy metals with abnormal deposition.

In this study, the limitations are mainly reflected in four points. Firstly, NGS data of tumor tissue DNA and ctDNA were acquired from single samples without matched normal tissue samples, because the actionable genomic alterations about the clinical decision are enough to get from a single sample according to the suitable filter conditions (Adzhubei et al. 2013; Genomes Project et al. 2015; Hiltemann et al. 2015; Kircher et al. 2014; McNulty et al. 2019; Ng and Henikoff 2003; Schwarz et al. 2010; Sukhai et al. 2019; Teer et al. 2017). What we cannot ignore is that the cost of multi-type or multiregional biopsies is much more than that of a single sample. Secondly, this study only examined the concentrations of heavy metals in serum, but did not elaborate on the effects of one metal with different forms, nor elaborate on the source of exposure (e.g., soil, food, water, or air). Thirdly, the concentrations of heavy metals were detected only in serum without the matched urine, hair, and nail. Hair and nails directly store heavy metals (e.g., Co, Cr, Cu, Fe, Ni, and Zn), which makes them appropriate for monitoring the effects of heavy metals on health. Thus, it is attractive to detect the concentrations of heavy metals in serum, hair, and nail samples from one person in future studies. Lastly, further studies with more sizeable sample sizes and multi-institution are necessary to validate the generalizability of our conclusion.

## Conclusions

In summary, the genomic alternations, somatic interactions, traditional serum biomarkers, and heavy metals were markedly different between patients with LUAC and LUSC, and heavy metals (e.g., Ba, Pb, and Cd) may contribute to the tumorigenesis of NSCLC with different histological and molecular subtypes.

## Supplementary Information

Below is the link to the electronic supplementary material.

Supplementary file1 (DOC 492 KB)

Supplementary file2 (XLSX 16 KB)

Supplementary file3 (XLSX 18 KB)

Supplementary file4 (XLSX 19 KB)

Supplementary file5 (XLSX 61 KB)

Supplementary file6 (XLSX 36 KB)

Supplementary file7 (XLSX 48 KB)

## Data Availability

The raw data supporting our conclusions are included in this article. The sequencing data are available at the NCBI BioProject database PRJNA904420.
