# Supplementary material for: Differences of genomic alterations and heavy metals in non-small cell lung cancer with different histological subtypes
Source: J Cancer Res Clin Oncol. 2023 May 31;149(12):9999–10013. doi: 10.1007/s00432-023-04929-2 (PMC10423170; doi:10.1007/s00432-023-04929-2)
Supplement: Supplementary file 1 — Supplementary file1 (DOC 492 KB) [file 432_2023_4929_MOESM1_ESM.doc]

**Differences of genomic alterations and heavy metals** **in non-small cell lung cancer with different histological subtypes**

Die Mu^1*^∙ Hui Tang^2^,^3*^ ∙ Gen Teng^1^ ∙ Xinyang Li^1^ ∙ Yarui Zhang^1^ ∙ Ge Gao^1^ ∙ Dongjuan Wang^1^ ∙ Lu Bai^1^ ∙ Xiangyao Lian^1^ ∙ Ming Wen^2^ ∙ Lisha Jiang^2^ ∙ Shouxin Wu^2^ ∙ Huihui Jiang^2^ ∙ Cuimin Zhu^1^


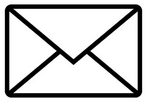
 Huihui Jiang

jianghh@biotecan.com


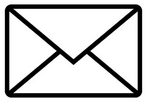
 Cuimin Zhu

minminzhu1006@163.com

**^*^**Contributed equally

^1^ Department of Oncology, Affiliated Hospital of Chengde Medical University, Chengde 067000, China

^2^ Shanghai Zhangjiang Institue of Medical Innovation, Shanghai Biotecan Pharmaceuticals Co., Ltd., Shanghai 200135, China

^3^ Department of Interventional and Vascular Surgery, Shanghai Tenth People's Hospital, School of Medicine, Tongji University, Shanghai 200072, China.


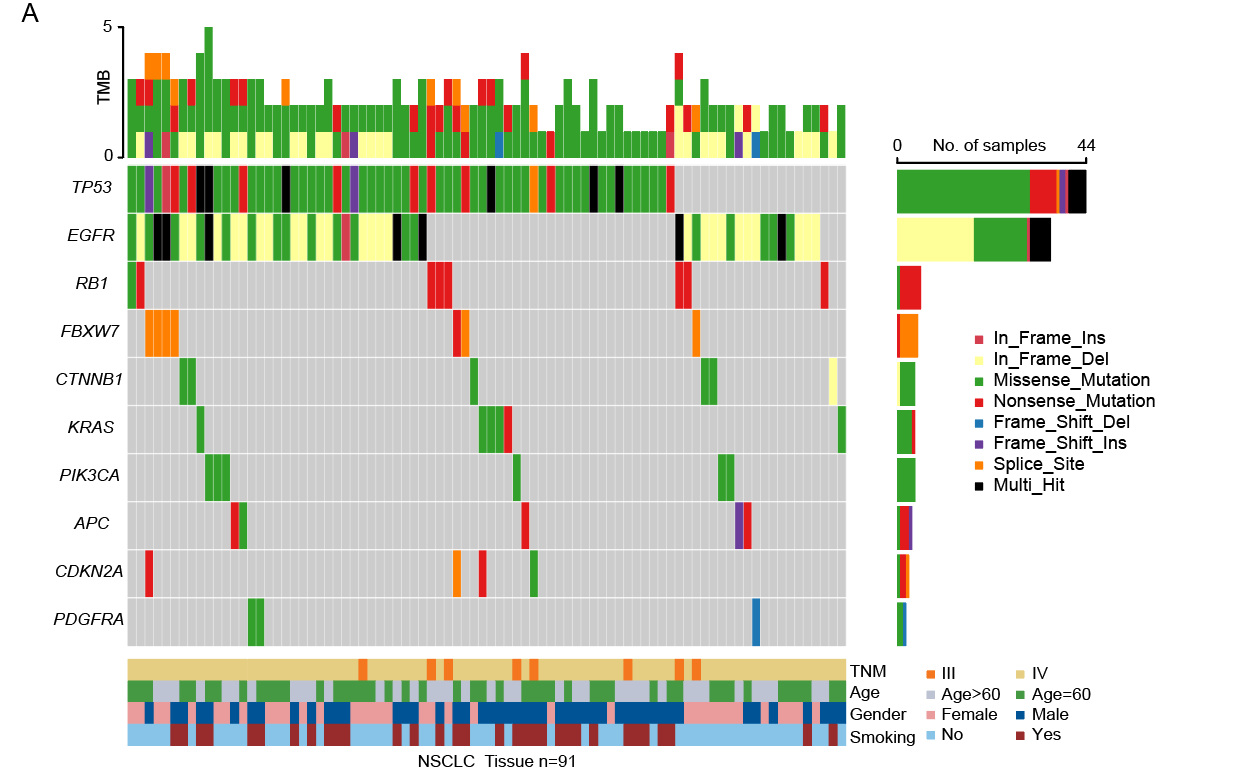

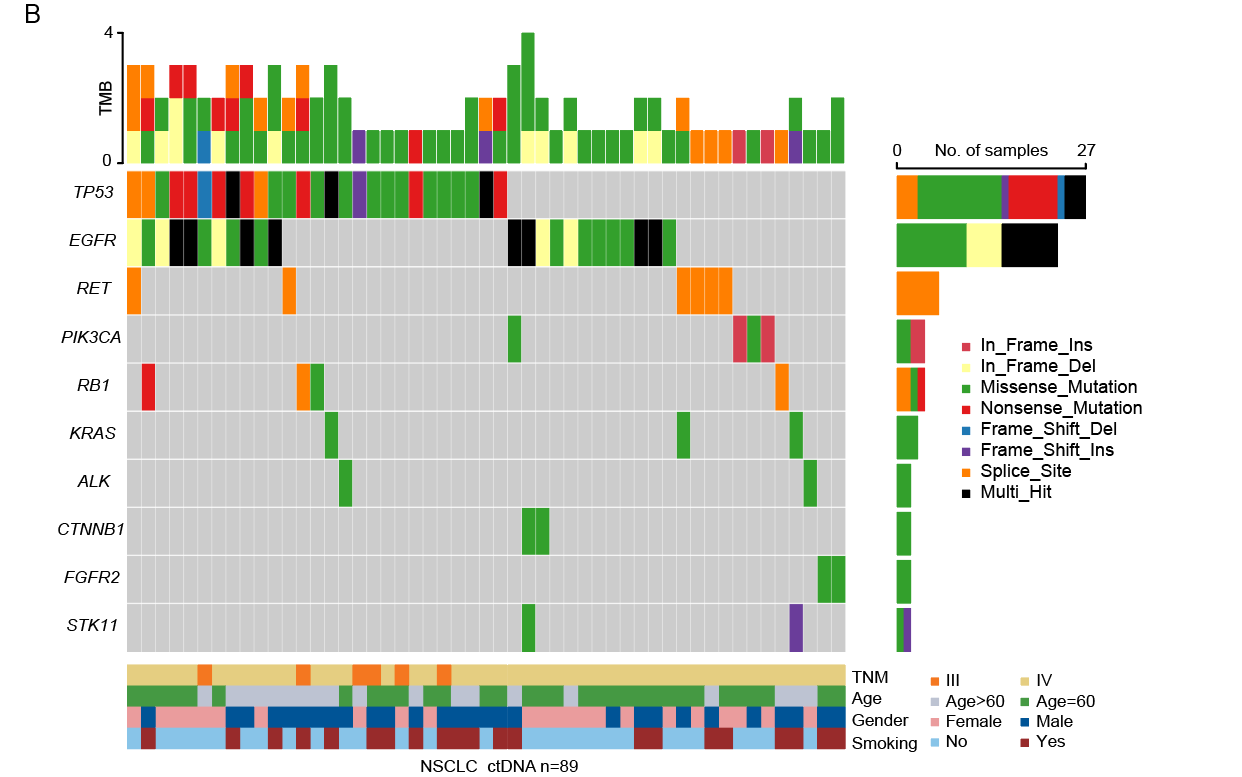

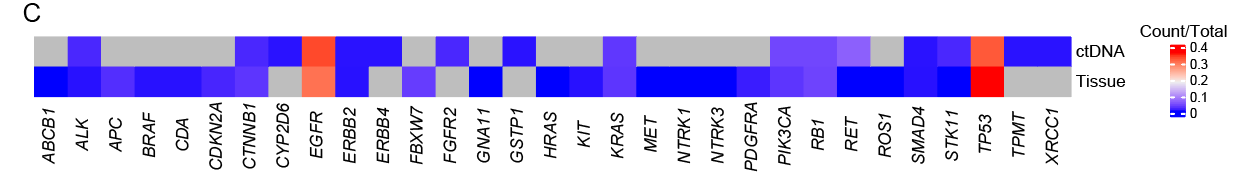


**Supplementary figure 1.** Somatic mutation landscape of NSCLC derived from tumor tissue DNA (n = 91) (A) and ctDNA (n = 89) (B). Patients were arranged along the x-axis. Mutant genes are ranked by mutant frequency and the right panel shows the number of samples with nonsynonymous mutations. Tumor mutation burden (TMB, mutations per Mb) is shown in the upper panel. Concordance of mutated genes derived from tumor tissue DNA or ctDNA in NSCLC patients (C).


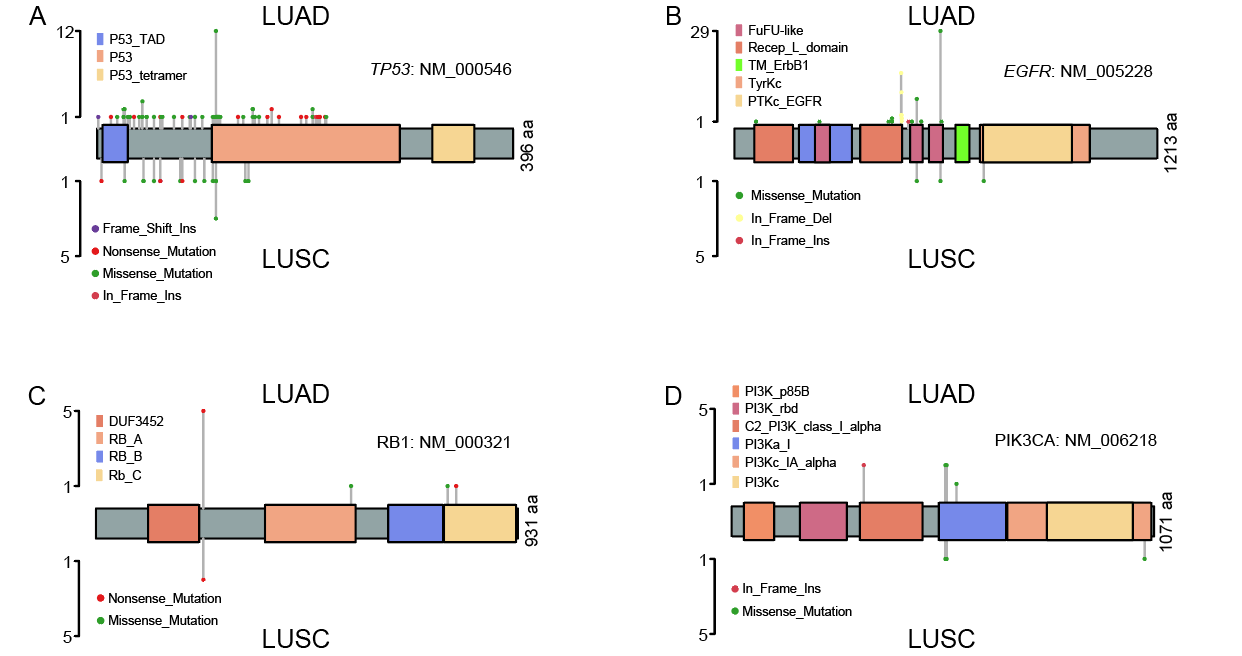

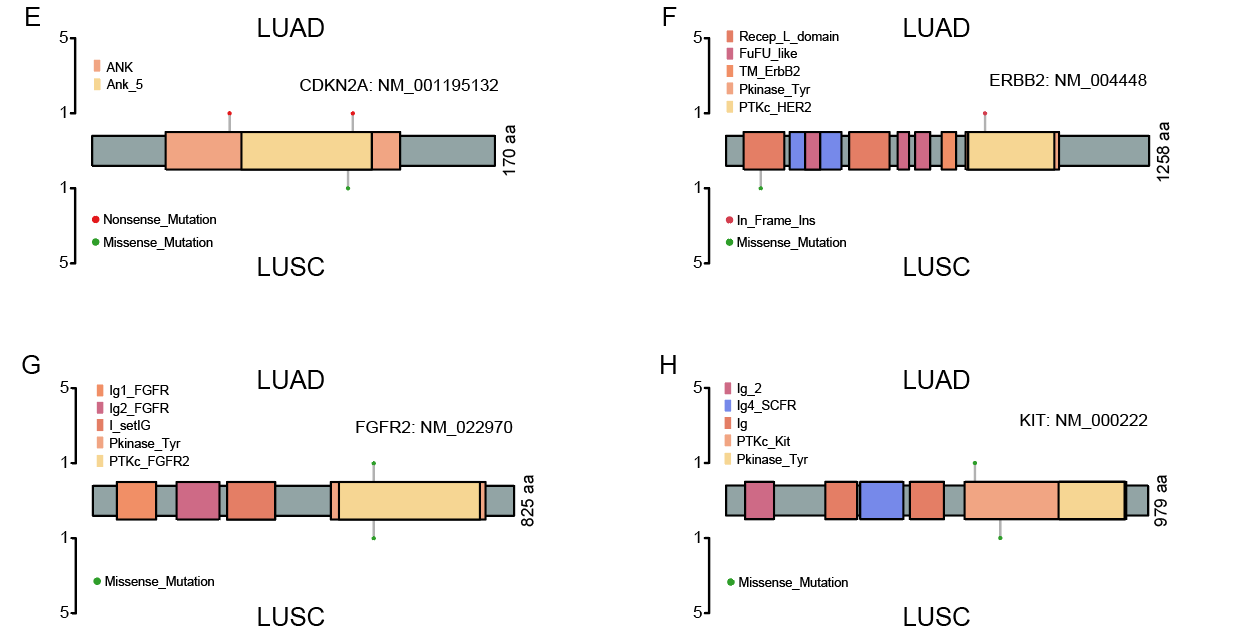


**Supplementary figure 2.** The protein structure and mutational proportion of TP53 (A), EGFR (B), RB1 (C), PIK3CA (D), CDKN2A (E), ERBB2 (F), FGFR2 (G), and KIT (H) between patients with LUAD (top panels) and LUSC (bottom panels). Protein domains are marked in different colors. Lollipops represent the locations of protein-altering variants. Square brackets indicate the proportion of patient-harbored non-synonymous mutations in each group.


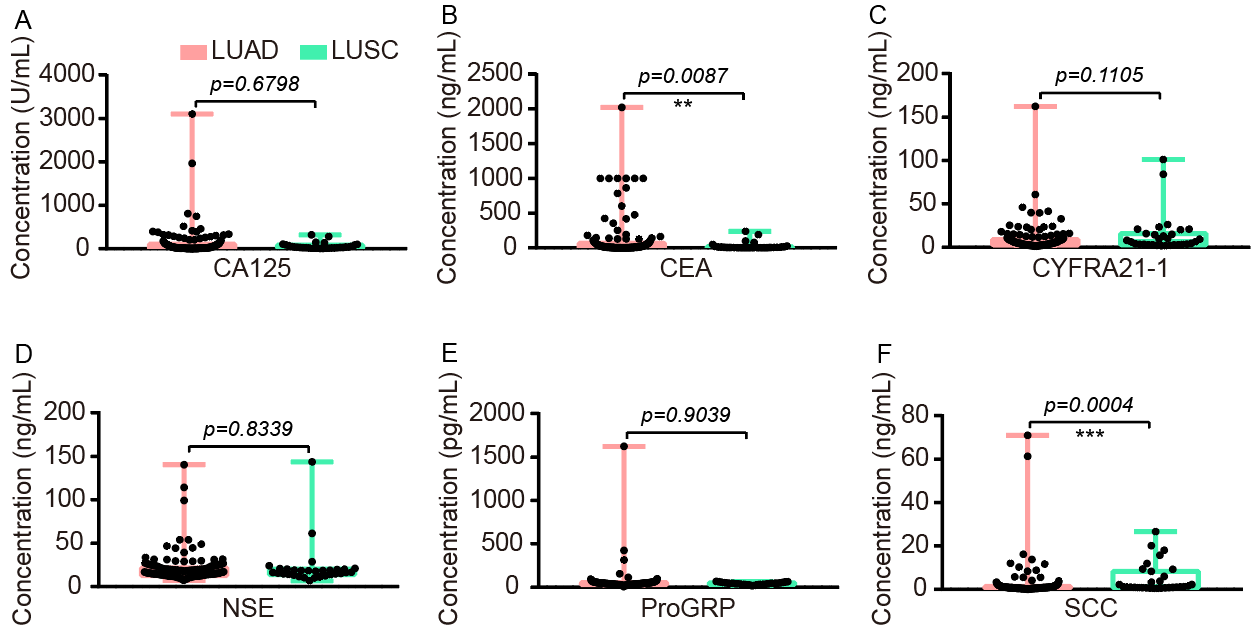


**Supplementary figure 3.** The comparative analysis of 6 traditional serum biomarkers (CA125, CEA, CYFRA21-1, NSE, ProGRP, and SCC) between patients with LUAD and with LUSC. Statistical analysis was performed by the Two-tailed Mann Whitney U test. ** p < 0.01, *** p < 0.001.


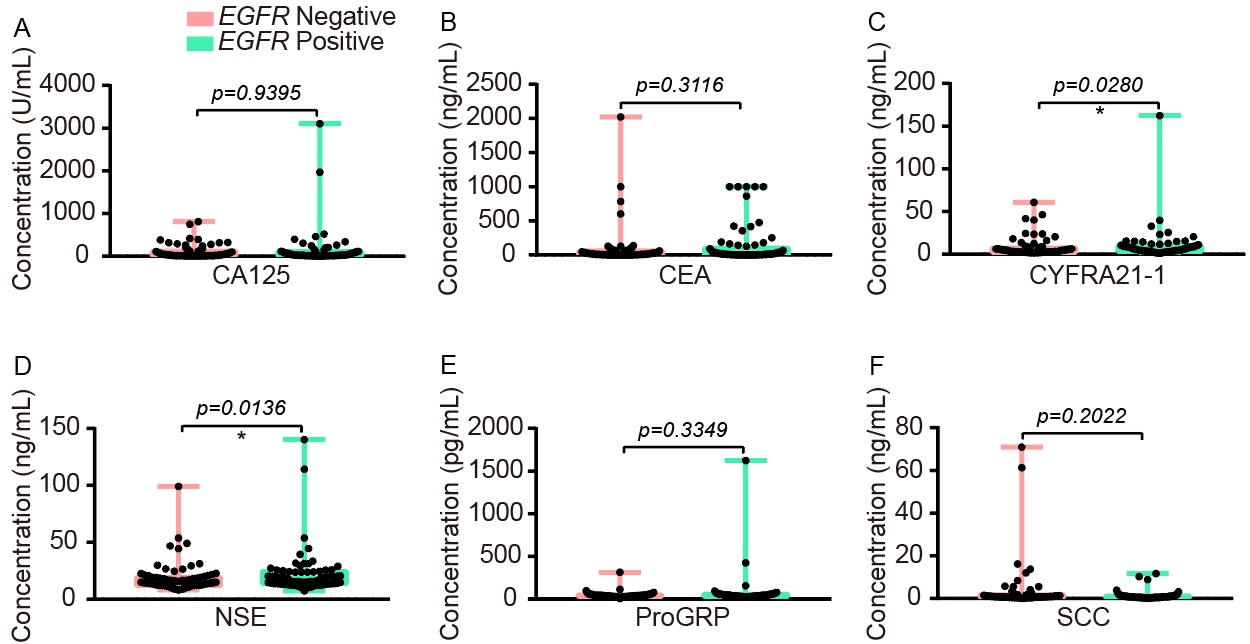


**Supplementary figure 4.** The comparative analysis of 6 traditional serum biomarkers (CA125, CEA, CYFRA21-1, NSE, ProGRP, and SCC) between LUAD patients with and without *EGFR* mutations. Statistical analysis was performed by the Two-tailed Mann Whitney U test. * p < 0.05.
